# Impact of a progressive stepped care approach in an improving access to psychological therapies service: An observational study

**DOI:** 10.1371/journal.pone.0214715

**Published:** 2019-04-09

**Authors:** Lisa Boyd, Emma Baker, Joe Reilly

**Affiliations:** 1 Tees Esk and Wear Valleys NHS Foundation Trust, Durham, England; 2 University of York, York, England; Universita degli Studi di Padova, ITALY

## Abstract

England’s national Improving Access to Psychological Therapies (IAPT) programme advocates stepped care as its organizational delivery of psychological therapies to common mental health problems. There is limited evidence regarding the efficacy of stepped care as a service delivery model, heterogeneity of definition and differences in model implementation in both research and routine practice, hence outcome comparison in terms of effectiveness of model is difficult. Despite sound evidence of the efficacy of low intensity interventions there appears to be a perpetuation of the notion that severity and complexity should only be treated by a high intensity intervention through the continuation of a stratified care model. Yet no psychotherapy treatment is found to be more superior to another, and not enough is known about what works for whom to aid the matching of treatment decision. In the absence of understanding precise treatment factors optimal for recovery, it may be useful to better understand the impact of a service delivery model, and whether different models achieve different outcomes. This study aims to contribute to the discussion regarding the stepped care definition and delivery, and explores the impact on clinical outcomes where different types of stepped care have been implemented within the same service. An observational cohort study analysed retrospective data (n = 16,723) over a 4 year period, in a single IAPT service, where delivery changed from one type of stepped care model to another. We compared the outcomes of treatment completers with a stratified care model and a progression care model. We also explored the assumption that patients who score severe on psychological measures, and therefore are potentially complex, would achieve better outcomes in a stratified model. Outcomes in each model type were compared, alongside baseline factor variables. A significant association was observed between a recovery outcome and model type, with patients 1.5 times more likely to recover in the progression delivery model. The potential implications are that with a progression stepped care model of service delivery, more patients can be treated with a lower intensity intervention, even with initial severe presentations, ensuring that only those that need high intensity CBT or equivalent are stepped up. This could provide services with an effective clinical model that is efficient and potentially more cost effective.

## Introduction

Depression and anxiety disorders are common mental health problems, with one in six people in their lifetime likely to suffer with a mental health problem at some point, with impacts on employment, relationships and general quality of life [1]. Investing in treatment for mental illness with a talking therapy may pay for itself by an increase in people working and reduction in the cost to the state [2, 3]. The evidence base for efficacy of psychological interventions in common mental disorders is well-established, however less is known about how to implement them. In addition, the notion that severe and complex presentations require a more intensive treatment pervades in service model design and clinical guidance. This is despite growing evidence of the efficacy of low intensity interventions [4]; not enough is known about what exactly works for whom.

The Improving Access to Psychological Therapies (IAPT) programme in England is a national service transformation and expansion development in place since 2008 [1]. By increasing resources and training to develop skill, it aims to increase access to psychological therapies for common mental disorders such as depression and anxiety on a large scale. Its goals are major clinical benefits, and also economic benefits in terms of return to work. Prescribed data is collated locally and nationally, which is useful for evaluation. The IAPT programme broadly takes a ‘stepped care’ approach. The stepped care model [5] is an organising framework of treatment and intensity recommended by the National Institute for Health and Clinical Excellence (NICE). NICE is a Non Departmental Public Body (NDPB) which is responsible for producing guidelines and recommendations for treatment using well researched evidence. The stepped care NICE guidelines set out which evidence based psychological treatments are recommended for which disorders. The National IAPT implementation guidelines [6] outline the expected treatment delivery including expected number of sessions to which disorder and it is assumed that all IAPT services are structured to follow this recommendation. The existing literature regarding stepped care defines it to be a service delivery system recommending the least intrusive intervention first, starting with low intensity treatments [7], with step up if no progress. Whilst the NICE guidelines do recommend least intrusive intervention first, they also recommend a higher intensity treatment in the first instance for more complex and severe presentations and specific disorders such as post-traumatic stress disorder (PTSD) and social anxiety, which is more akin to a stratified model. The stratified model is a form of stepped care where choice of a lower or higher intensity treatment is made by the assessing therapist, in contrast to a progressive stepped care approach in which there is provision of low intensity treatment first. IAPT guidance recommends that service delivery follows NICE guidelines [6] where there are a range of prescribed treatments offered at a step 2, low intensity and step 3 high intensity levels for specific disorders. It is assumed that all IAPT sites will be following the national IAPT implementation guidelines and delivering a version of stepped care, but with variation between emphasis on stratified and progressive models across services. One study demonstrates this with an evaluation of the development of four mental health service sites [8]. All developed a slightly different interpretation of stepped care to each other, demand and capacity was seen to influence service design, as was clinician bias effecting pathway flow. Systems for psychological treatment delivery are less well-researched than therapies themselves, and evidence of the effectiveness of stepped care is limited, particularly when comparison of stratified and progressive models is sought. Two recent systematic reviews [9, 10] found considerable heterogeneity between studies on types of treatment, and method of step up. Both concluded that in general, stepped care can be seen as effective when compared with usual care. Limitations included substantial heterogeneity between studies and underpowered samples.

Underpinning the apparent preference in the literature, routine practice and clinical guidelines for a stratified or mixed model of stepped care, appears to be the notion that the more complex and severe the presentation, a higher intensity treatment is needed. This is not supported by evidence. In a recent meta-analysis [4], severe presentations had as much clinical gain with low intensity treatments as those less severe. Another study compared outcomes of low and high intensity treatment and found no difference in baseline scores between low or high intensity treatments [11]. Similarly, a different study [12] found initial scores were not an influencing factor on a service achieving low or high recovery rates. A systematic review [10] found considerable variation of patient severity and symptom chronicity and no clear trends that related chronicity/severity to clinical outcome. Furthermore, the approach of stratifying care by severity assumes we may know ‘what works for whom’, whereas a recent network meta-analysis found there to be no superiority between therapy modality [13].

In designing or evolving psychological therapy service delivery for common mental disorders, it would be extremely useful to have systematic evidence on how a progressive model of stepped care compares to a stratified model. Examining this question via a randomised controlled trial is challenging, perhaps requiring a cluster method and very large samples. However, the opportunity now exists in England to examine a large observational dataset of patient characteristics and outcomes within IAPT services to study associations between service models and outcomes.

This paper describes an observational study using retrospective data in routine practice. The study compares service delivery of stratified care, and progressive stepped care within a single IAPT service, taking the opportunity of a natural experiment due to model change over time. Two main research questions are addressed:

1. What is the relationship between clinical outcomes of depression and anxiety and service delivery model for adults treated in an IAPT service?
2. What is the relationship between the clinical outcomes for moderate to severe anxiety and depression, and service delivery model?

The relationship with outcome and model and baseline variables is also explored.

## Method

### Ethics statement

A study proposal outlining the methodology, and that the retrospective data would be anonymised at source was submitted to Durham University ethics committee and was approved. The proposal was also discussed and approved by Tees, Esk and Wear Valleys NHS Foundation Trust Research and Development Department (R&D) and the Trust’s Quality Assurance group.

### Study site

The study site is a large IAPT service in North East of England with at the time around 90 clinical therapists, either trainee or qualified as Psychological Wellbeing Practitioners (low intensity interventions step 2) or High Intensity Therapists (E.g. Cognitive behavioural therapy or interpersonal therapy delivered at step 3). IAPT services across England generally deliver interventions at step 2 and 3 of the stepped care model outlined by NICE [5]. During the first two years of delivery, the study site operated mainly with a stratified model of stepped care. The decision making point for treatment level occurred after assessment, Those deemed severe or complex by the assessing clinician were placed straight onto step 3 high intensity waiting lists, and those initially treated at step 2 were stepped up if needed. By the end of year one the waiting lists at step 3 were unacceptably long, with underused capacity at step 2. A decision was made during year 2 to move towards a progression model of stepped care; informed by a growing body of evidence regarding the efficacy of low intensity interventions, even with severe presentations [4]. This offered a potentially efficient, cost effective and evidence based solution to the growing waiting list at step 3. A pragmatic decision was taken regarding presentations of PTSD and social anxiety; given the waiting list issues at the time. It was believed that to offer psycho educative support through step 2 low intensity interventions in the first instance and then stepped up would be more helpful than receiving no treatment, or waiting a long time, and aided clinical risk management.

In terms of robustness of data analysis regarding model delivery, years 2 and years 4 were isolated to minimise variable effect of poorer data input in year 1 as systems refined, and model changeover effect in year 3.

As with all IAPT services, the study site submits throughput and outcome data for national evaluation. A minimum dataset (MDS) is collected at each assessment and intervention session, compromising of a number of psychological measures and demographic information, including ethnicity, disability and employment status as defined by the national IAPT programme [14]. The data is evaluated against a specific set of key performance indicators (KPI’s). Clinical recovery is defined through movement from above to below certain scores on two psychological measures, PHQ9 and GAD7.

### Patients

16,723 patients were included in the initial analysis. These were patients who had been assessed, completed treatment and discharged by the service within the four year period. The definition of completed treatment used in this study (apart from a comparator in discharge reasons) was that used for the national IAPT KPI’s. Completed treatment is defined as two or more separate treatment sessions.

### Design

An observational retrospective cohort design was used. Four years of routine data was extracted for the purposes of specific evaluation. Descriptive analysis was undertaken on the whole dataset (n = 16,723). Year 2 and 4 data were then isolated (n = 8578), to control the effect of contaminated or less robust data from service set up and system refinement during year 1, and model delivery changeover in year 3. Year 2 (n = 3932) represents a stratified model of delivery, and year 4 (n = 4646) a progression model. Subset cohorts were also selected using moderate to severe initial scores of the psychological measures. (Years 2&4, PHQ9 moderate to severe initial scores n = 5440, GAD7 moderate to severe initial scores n = 7398).

As described earlier, treatments offered by IAPT services are prescribed by the national IAPT implementation programme, which uses NICE guidelines. Therefore the effectiveness of such treatments are already well researched, agreed by NICE, and are not within the scope of this study to measure. Types of psychological treatment delivered were not a controlled variable in this study. Within the progression model, some patients potentially with PTSD or social anxiety may have received a step 2 intervention in the first instance. Initial identification of these patients may have proved difficult to isolate as at the time of the study, as the service needed to improve its initial disorder coding within the clinical database. Also it is possible that definite diagnosis of these presentations may occur at a later stage during therapeutic intervention, when the patient discloses at a later session rather than at initial assessment.

As therapists employed within IAPT are required to be trained and qualified in specific therapeutic treatment delivery, there is the assumption that NICE and IAPT guidelines as described earlier are adhered to. The service in this study ensured regular clinical supervision and clinical records audit as part of clinical governance to provide assurance regarding treatment fidelity. Whilst it is accepted that there may be some variation of fidelity to treatment model, this study concerns routine practice data, with the likelihood of co-morbid presentations and therefore it would be expected that treatment will appropriately vary to reflect this. The outcomes of IAPT services against NICE compliance regarding treatment has already been measured [15] and found compliance with NICE guidance was associated with higher recovery rates. This study is not a randomised controlled trial (RCT) and it is not within the scope to measure treatment fidelity.

In terms of treatment dosage, the service within this study delivered manualised sessions at step 2, e.g. 4 sessions for telephone guided self-help or psycho education course, and 6–8 sessions face to face guided self-help. Step 3 is less prescriptive, advocating the clinician to follow the NICE guidelines of between 12–16 sessions on average.

### Psychological measures and outcomes

The minimum data set (MDS) includes a number of measures, however for the purposes of this study the data from the specific phobia questions and the work and social adjustment scale were not analysed. The specific measures counted in this study are the PHQ-9 and GAD-7. The PHQ-9 is a nine question scale that measures depression symptoms frequency scoring from 0, “not at all bothered by the problem”, to 27 “bothered nearly every day”. The reliability and validity of the PHQ-9 in terms of measuring depression is good [16]. The GAD-7 is a seven question scale that measures the frequency of anxiety symptoms scoring from 0–21. The reliability and validity of the GAD-7 in terms of measuring general anxiety symptoms is good, and satisfactory with more specific disorders such as social phobia, or obsessive compulsive disorder [17]. The scales are used in every clinical session and the scores at the first and last sessions are used to measure outcome. The KPI defines recovery for IAPT as patients scoring above clinical caseness at first session on at least one measure, and below caseness on both measures at the last session to count as recovered (caseness = 10 on PHQ-9, 8 on GAD-7). Reliable clinical change was defined in the same way as another large IAPT study [15] which outlines; “Patients were deemed to have reliably recovered if they scored above the clinical cut-off on the PHQ-9 and/or the GAD-7 at initial assessment, they showed reliable improvement during treatment, and they scored below the clinical cut-offs on both the PHQ-9 and the GAD-7 at the end of treatment. Reliable improvement was assessed using Jacobson and Truax (1991) reliable change criteria. The measure of reliability used for the PHQ-9 and the GAD-7 was Cronbachs α, taken from the validation studies of the measures (Kroenke et al., 2001; Spitzer et al., 2006).”

### Statistical analysis

Statistical analysis was performed using the software SPSS version 20. A statistician was consulted and recommended the use of cross tabulations to measure proportion and frequency, chi square tests–two sided, to show any association between categorical variables, and multiple logistic regression to test variables as predictors of outcomes. Variables were entered simultaneously. The value of *p* was set at 0.05 in terms of significance for all tests. The dataset preparation for multiple logistic regression removed outliers and certain variables.

Given extremely low numbers in both ends of the age categories, those were removed. The variables ethnicity and disorder type were also removed. The ethnicity variation other than White British was not of a number to be able to undertake further statistical analysis.

The disorder type as a variable was removed for the regression tests as there was not enough confidence that this data represented an accurate picture of patient presentation. In the years that this data applies to, the information was taken from the referral at the point of receipt, prior to assessment. It was believed that there may be a large number where the disorder category, i.e. the primary reason for treatment may change after assessment and indeed perhaps at a later point after some therapy sessions. In particular the nature of PTSD may mean initial presentation of depression or anxiety but a disclosure at a later point reveals the actual disorder. It was felt that it would not be helpful to include this variable and make conclusions particularly on specific disorders given the amount of potential variation described. It is acknowledged by doing so; a comment cannot be made regarding the service delivery model impact on clinical outcomes regarding disorder type, other than acknowledging disorder type may be a confounding variable that at this stage was difficult to control. Subsequent developments with IAPT services mean that it is possible to now update disorder type within a clinical database to reflect a more accurate picture. Also, the nature of this study uses routine practice data, the study is not an RCT. Therefore it is perhaps more applicable to consider that there will be large elements of co-morbidity within the dataset, and that the study is concerned with measuring the broad impact of service model delivery on clinical outcomes for a routine clinical population.

The initial regression used the dependent variable (outcomes) arranged as binary, i.e. recovered versus non-recovered. Independent variables included the model delivery type, stratified or progression, initial severity scores, patient characteristics and discharge reasons.

The stratified model type was the reference. Initial severity scores were grouped into categories using the severity categories defined in the IAPT data handbook [14] which enabled them to be categorical variables and are more meaningful to compare the results. Demographic variable categories were used in line with national IAPT KPI reporting, as described earlier with regards to gender, disability and ethnicity. Age was organised into categorical variables using mainly 10 year age groupings aligned with population reports undertaken by the Office of National Statistics.

Discharge reasons were service defined categories; completed treatment, dropped out, not suitable, declined treatment and referred on. These are chosen by the clinician and entered into the clinical database at point of patient discharge. Completed treatment defined by clinician as a discharge reason is different from completed treatment defined by IAPT KPI definition as described earlier. Completed treatment IAPT KPI definition was used to select the whole dataset. Looking at therapist defined completed treatment as a discharge reason enables comparison with other discharge reasons i.e. dropped out.

To explore the question regarding the impact of service delivery model on presentation severity and outcome, regression tests were undertaken with the moderate to severe initial score groups.

## Results

The descriptive analysis showed a normal distribution of the clinical population of this study site, regarding demographics and psychological measures, comparable to the national IAPT data [18]. Chi square tests showed a significant association between a number of independent variables, and between model type and recovery. The volume of patients being treated and completing treatment rose incrementally from 1893 in year one, to 4291 in year 2, and 5145 in year 4. As the progression model was introduced, the numbers being treated at step 2 in the first instance increased, with a proportional reduction of those treated at step 3 in the first instance in year 4 at 7.6%, compared to year 2 at 23%.

### Baseline factors

There was no cohort difference in the spread of baseline factors. Descriptive analysis of the whole dataset (n = 16,723) in Table 1 showed a mean age of 42.3 years, (SD = 13.9, Mdn = 42, IQR = 21). There were a larger proportion of younger women than men, with the difference in gender decreasing as the age bands rise. 28% of men were unemployed compared to 19% women. 13% men and 10% women are categorised sick or disabled. Within those registered disabled, there were more men than women registered with a significant association found between gender and disability, X^2^ (1, N = 16,718) = 31.7, p = <0.001). There were also more in the age bands 45–54, and 55–64, with a larger proportion being male.

**Table 1:** 4 Years dataset: Demographics by year.

		Year One		Year Two		Year Three		Year Four		Total	
		N	%	N	%	N	%	N	%	N	%
Gender	Male	702	37.1	1590	37,1	2058	38.2	1867	36.3	6217	37.2
	Female	1191	62.9	2699	62.9	3333	61.8	3278	63.7	10501	62.8
	Not Stated	0	0	2	0	3	0.1	0	0	5	0
	Total	1893	11.3	4291	25.7	5394	32.3	5145	30.8	16723	100
Age	16–17	0	0	0	0	0	0	3	0.1	3	0
	18–24	90	4.8	315	7.3	554	10.3	705	13.7	1664	10
	25–34	447	23.6	1019	23.7	1256	23.3	1211	23.5	3933	23.5
	35–44	482	25.5	983	22.9	1220	22.6	1180	22.9	3865	23.1
	45–54	435	23	991	23.1	1182	21.9	1086	21.1	3694	22.1
	55–64	297	15.7	689	16.1	866	16.1	670	13	2522	15.1
	65–74	116	6.1	242	5.6	249	4.6	218	4.2	825	4.9
	75–84	21	1.1	45	1	55	1	60	1.2	181	1.1
	85–94	5	0.3	7	0.2	12	0.2	11	0.2	35	0.2
	95+	0	0	0	0	0	0	1	0	1	0
	Total	1893	11.3	4291	25.7	5394	32.3	5145	30.8	16723	100
Ethnicity	British	685	36.2	2564	59.8	4741	87.9	4809	93.5	12799	76.5
	Irish	1	0.1	3	0.1	7	0.1	7	0.1	18	0.1
	Other White background	3	0.2	15	0.3	33	0.6	50	1	101	0.6
	White & Black Caribbean	2	0.1	1	0	13	0.2	7	0.1	23	0.1
	White & Black African	0	0	1	0	0	0	1	0	2	0
	White & Asian	0	0	2	0	5	0.1	1	0	8	0
	Other mixed background	1	0.1	2	0	7	0.1	6	0.1	16	0.1
	Indian	2	0.1	10	0.2	18	0.3	14	0.3	44	0.3
	Pakistani	0	0	1	0	3	0.1	4	0.1	8	0
	Bangladeshi	0	0	1	0	1	0	0	0	2	0
	Other Asian background	0	0	3	0.1	8	0.1	7	0.1	18	0.1
	Caribbean	1	0.1	1	0	2	0	5	0.1	9	0.1
	African	1	0.1	0	0	4	0.1	2	0	7	0
	Other Black background	3	0.2	1	0	3	0.1	0	0	7	0
	Chinese	0	0	9	0.2	21	0.4	11	0.2	14	0.1
	Other ethnic group	4	0	3	0.1	7	0.1	15	0.3	56	0.3
	Not Stated	1190	62.9	1674	39	521	9.7	206	4	3591	20.9
	Total	1893	11.3	4291	25.7	5394	32.3	5145	30.8	16723	100
Disability	Yes	61	3.2	185	4.3	536	9.9	494	9.6	1276	7.6
	No	1832	96.8	4106	95.7	4858	90.1	4651	90.4	15447	92.4
	Total	1893	11.3	4291	25.7	5394	32.3	5145	30.8	16723	100
Referred Problem	Mental & Behavioural Disorder due to alcohol use	1	0.1	1	0	4	0.1	3	0.1	9	0.1
	Bipolar Affective Disorder	0	0	2	0	6	0.1	8	0.2	16	0.1
	Depressive Episode	437	23.1	939	21.9	1362	25.3	1144	22.2	3882	23.2
	Recurrent Depressive Episode	142	7.5	330	7.7	389	7.2	273	5.3	1134	6.8
	Dysthymia		0		0	5	0.1	7	0.1	12	0.1
	Agoraphobia	16	0.8	32	0.7	31	0.6	24	0.5	103	0.6
	Social Phobias	24	1.3	21	0.5	64	1.2	71	1.4	180	1.1
	Specific Phobias	26	1.4	33	0.8	65	1.2	49	1	173	1
	Panic Disorder	38	2	195	4.5	293	5.4	229	4.5	755	4.5
	Generalized Anxiety Disorder	522	27.6	1130	26.3	999	18.5	664	12.9	3315	19.8
	Mixed Anxiety & Depressive Disorder	507	26.8	1310	30.5	1625	30.1	2037	39.6	5479	32.8
	Obsessive Compulsive Disorder	33	1.7	104	2.4	182	3.4	143	2.8	462	2.8
	Post Traumatic Stress Disorder	24	1.3	59	1.4	158	2.9	144	2.8	385	2.3
	Adjustment Disorder	1	0.1	3	0.1	35	0.6	40	0.8	79	0.5
	Somatoform Disorder	1	0.1	1	0	22	0.4	25	0.5	49	0.3
	Hypochondriac Disorder	3	0.2	3	0.1	3	0.1	3	0.1	12	0.1
	Eating Disorder	5	0.3	26	0.6	34	0.6	24	0.5	89	0.5
	Other Mental Disorder	63	3.3	91	2.1	103	1.9	250	4.9	507	3
	Disappearance or Death of Family Member	2	0.1	0	0	14	0.3	7	0.1	23	0.1
	Not Stated	48	2.5	11	0.3	0	0	0	0	59	0.4
	Total	1893	11.3	4291	25.7	5394	32.3	5145	30.8	16723	100
Employment Status	Employed	922	48.7	2011	46.9	2548	47.2	2455	47.7	7936	47.5
	Unemployed & Seeking Work	511	27	1054	24.6	1192	22.1	950	18.5	3707	22.2
	Students Not Seeking Work	92	4.9	269	6.3	361	6.7	378	7.3	1100	6.6
	Sick or Disabled	127	6.7	399	9.3	617	11.4	720	14	1863	11.1
	Homemaker	90	4.8	258	6	287	5.3	267	5.2	902	5.4
	No Benefits, Not Working or Seeking Work		0	5	0.1	20	0.4	12	0.2	37	0.2
	Voluntary Work		0	1	0	3	0.1	3	0.1	7	0
	Retired	121	6.4	269	6.3	342	6.3	329	6.4	1061	6.3
	Not Stated	30	1.6	25	0.6	24	0.4	31	0.6	110	0.7
	Total	1893	11.3	4291	25.7	5394	32.3	5145	30.8	16723	100

### Impact of model type on outcomes

In terms of the mean recovery rates, there is a distinct difference of 9% between the models with the stratified model measuring 40% as recovered, compared to the progression model measuring 49% as recovered. Chi square tests showed an association between recovered versus non recovered outcome and model, *X*^*2*^ (1, *N* = 8578) = 78.6, *p* = <0.001, No correlation was found for reliable improvement or reliable deterioration.

Treatment results as demonstrated in Table 2 showed there was a small increase in treatment dosage within the progression model compared to the stratified.

**Table 2:** Mean treatment dosage.

	Stratified		Progression	
	Average Number of Sessions	Number of patients (%)	Average Number of Sessions	Number of patients (%)
Step 2 only	4.8	58.70	4.9	64.20
Step 3 only	9.2	27.30	10.2	10.80
Stepped up	10.9	12.30	12.8	23.40
Stepped down	6.8	1.80	6.5	1.60

Reasons for discharge were analysed and as shown in Table 3 there was nothing notable between model delivery type in terms of the various reasons for discharge such as not suitable, referred on, and declined, however there was an increase in therapist defined completed treatment in the progression model, and a small decrease in dropout rate within the progression model compared to the stratified model.

**Table 3:** Treatment completion by model type.

	Stratified		Progression	
	Number	%	Number	%
Completed Treatment	2555	59.50	3289	63.90
Dropped Out of Treatment	930	21.70	1005	19.50
Other	806	18.80	851	16.60

Note: As defined by therapist.

Logistic regression showed that patients were 1.53 times more likely to recover in the progression model, as shown in Table 4. (Wald statistic (1) = 57.075, *p<* .001, OR = 1.53, lower CI = 1.368, upper CI = 1.705). The data were shown to fit the model using the Hosmer and Lemeshow test (*p* ˃ .05). The logistic regression model was statistically significant, *X*^*2*^(25) = 3555.72, *p* < .001. The model explained 45.6% of the variance in the recovery outcome (using Nagel-kerke’s *R*^*2*^) and correctly classified 76.5% of the cases. Sensitivity was 79.8%, specificity was 73.7%. The positive predictor value was 72.1% and the negative predictor value was 81%.

**Table 4:** Logistic regression investigating the impact of service model type on recovery outcome and demographic variables.

	B	S.E	wald	df	Sig	OR	95% C.I FOR OR	
							Lower	Upper
MODEL(1)	0.42	0.06	57.1	1	<0.001	1.527	1.368	1.705
GENDER(1)	-0	0.06	0.18	1	0.675	0.976	0.872	1.093
DISABILTY(1)	-0	0.11	0.05	1	0.818	0.975	0.787	1.209
AGE			30.3	5	<0.5			
Age 25–34	0.09	0.11	0.63	1	0.428	1.093	0.877	1.364
Age 35–44	0.23	0.12	3.77	1	0.052	1.255	0.998	1.578
Age 45–54	2.29	0.12	5.84	1	0.016	1.332	1.056	1.681
Age 55–64	0.18	0.13	1.91	1	0.167	1.192	0.929	1.53
Age 65–74	1.01	0.21	23.5	1	<0.001	2.745	1.825	4.129
EMPLOYMENT			161	5	<0.5			
Unemployed	-0.7	0.07	99.3	1	<0.001	0.485	0.42	0.559
Student	-0.3	0.13	4.06	1	0.044	0.771	0.599	0.993
Sick/Disabled	-0.9	0.09	96.3	1	<0.001	0.409	0.342	0.489
Homemaker	-0.5	0.12	16.8	1	<0.001	0.614	0.486	0.775
Retired	-0.7	0.18	16.3	1	<0.001	0.482	0.338	0.687
PHQ			152	4	<0.5			
PHQ minimal	-0.2	0.22	0.5	1	0.479	0.853	0.55	1.324
PHQ moderate	-0.7	0.21	9.82	1	0.002	0.516	0.342	0.781
PHQ mod to severe	-1	0.21	24.2	1	<0.001	0.356	0.235	0.537
PHQ severe	-1.3	0.21	39.6	1	<0.001	0.262	0.173	0.398
GAD			125	3	<0.5			
GAD minimal	0.06	0.24	0.06	1	0.815	1.059	0.656	1.709
GAD moderate	-0.5	0.24	3.74	1	0.053	0.634	0.4	1.006
GAD severe	-914	0.24	15.2	1	<0.001	0.401	0.253	0.635
Discharge Reasons			1548	5	<0.001			
Completed	-2.8	0.09	1062	1	<0.001	0.062	0.053	0.074
Dropped out	-2.4	0.21	139	1	<0.001	0.088	0.059	0.131
Not suitable	-2.1	0.12	300	1	<0.001	0.12	0.094	0.152
Declined treatment	3.43	0.22	251	1	<0.001	0.032	0.021	0.049
Referred on	-1.3	0.01	89.8	1	<0.001	0.265	0.201	0.349
Constant	2.18	0.33	44.2	1	8.853			

Key: reference groups: Model type—stratified. Gender—female. Disability—no.

### Impact of relationship between patient characteristics and model type on outcomes

Within the logistic regression model where the progression service design was shown to be more likely to attain recovery than the stratified design, gender and disability were not found to affect the recovery outcome. Certain ages were found to affect the recovery outcome, with patients aged 44–54 1.33 times more likely to recover than the group 18–24 (*p* = 0.016). The 65–74 group were 2.75 more likely to recover than the 18–24 group (*p* = < .001). Patients in employment were also more likely to recover.

### Initial score severity impact

The median initial score for PHQ-9 was 16, GAD-7 median was 15, (Table 5) placing the most common score within the severe range for both measures. The last scores median were 8 for PHQ-9, and 7 for GAD-7, placing the most common within the below caseness range on both measures.

**Table 5:** Initial analysis of psychological measure scores.

	Stratified				Progression			
	Median	IR	Mean	SD	Median	IR	Mean	SD
PHQ First	16	10	15.7	6.3	16	9	15.4	6.2
PHQ Last	9	12	10.1	7.4	7	11	9.1	7.1
GAD First	15	8	13.9	5.1	15	7	14.0	5.0
GAD Last	8	10	9.0	6.4	7	10	8.2	6.1

The stratified and progression model data were then also separated by score severity using the groups described earlier. Cross tabulations and chi square tests were undertaken on the higher PHQ-9 and GAD-7 groups and show an association between score severity, outcome and model, a larger proportion of participants with moderate (GAD7), moderate/severe (PHQ9), or severe (GAD7 or PHQ9) initial scores recovered in the progression model compared to stratified, with significant association.

Chi square tests were undertaken on the higher PHQ9 groups and show an association between score severity, outcome and model. Severe PHQ9 showed *X*^*2*^ (3, *N* = 2776) = 27.2 *p =* <0.001, with participants scoring severe a larger proportion recovered in progression model, a larger proportion did not recover in stratified and there was no discernible difference with reliable improvement or reliable deterioration. Moderate/severe PHQ9 showed *X*^*2*^ (3, *N* = 2664) = 30.1, *p* = <0.001, with participants scoring moderate to severe a larger proportion recovered in year 4, (10% proportional difference between years) and a larger proportion attained reliable improvement, reliable deterioration or did not recover in the stratified model.

Cross tabulations and chi square tests were undertaken on the higher GAD7 score groups and showed an association between severity, outcome and model. The GAD7 severe group showed *X*^*2*^ (3, *N* = 4759) = 40.7, *p* = <0.001, with the severe group the largest proportion to recover was in the progression model than in stratified (over 8% proportional difference between years), and conversely a larger proportion of reliable improvement, reliable deterioration, and non-recovery in stratified. The moderate GAD 7 group showed *X*^*2*^ (3, *N* = 2639) = 24.8, *p* = <0.001, with the largest proportion to recover in progression model, with over 10% difference between years. The stratified model has the larger proportions for reliable improvement, reliable deterioration and non-recovery.

Logistic regression was undertaken with the score severity cohort (Table 6). The data were shown to be a good logistic regression model fit with the moderate to severe groups of initial scores of PHQ9 and GAD7, and all statistically significant (*p* < .001) more likely to recover in the progression model. The odds ratios were 1.28 for moderate PHQ9, 1.79 for moderate/severe and 1.6 with severe PHQ9. The odds ratios for GAD7 were moderate 1.49, and severe 1.55.

**Table 6:** Summary of logistic regression of impact of service model type on severity score group outcomes.

		B	S.E	wald	Sig	OR	95% C.I FOR EXP(B)	
							Lower	Upper
PHQ Moderate	Recovered	0.244	0.111	4.85	0.28	1.277	1.027	1.587
	Reliable Improvement	-0.016	0.141	0.012	0.912	0.985	0.747	1.298
	No change	-0.249	0.121	4.218	0.04	0.78	0.615	0.989
	Reliable Deterioration	-0.069	0.2	0.119	0.731	0.933	0.63	1.382
PHQ Moderate/Severe	Recovered	0.584	0.098	35.49	<0.001	1.794	1.48	2.174
	Reliable Improvement	-0.346	0.093	13.74	<0.001	0.708	0.589	0.85
	No Change	-0.128	0.105	1.486	0.223	0.88	0.717	1.081
	Reliable Deterioration	-0.44	0.205	4.619	0.032	0.644	0.431	0.962
PHQ Severe	Recovered	0.474	0.105	20.47	<0.001	1.606	1.308	1.972
	Reliable Improvement	-0.054	0.082	0.431	0.512	0.948	0.808	1.112
	No change	-0.311	0.094	10.89	0.001	0.733	0.609	0.882
	Reliable Deterioration	-0.196	0.282	0.485	0.486	0.822	0.473	1.427
GAD 7 Moderate	Recovered	0.4	0.099	16.17	<0.001	1.491	1.227	1.812
	Reliable Improvement	-0.25	0.115	4.739	0.029	0.778	0.621	0.975
	No change	-0.258	0.106	5.887	0.015	0.773	0.628	0.952
	Reliable Deterioration	0.049	0.18	0.075	0.784	1.051	0.738	1.496
GAD 7 Severe	Recovered	0.436	0.076	33	<0.001	1.546	1.333	1.794
	Reliable Improvement	-0.101	0.064	2.504	0.114	0.904	0.798	1.024
	No Change	-0.234	0.075	9.672	0.002	0.791	0.683	0.917
	Reliable Deterioration	-0.532	0.218	5.944	0.015	0.587	0.383	0.901

Note: the reference group for the model type is the stratified model, therefore the values shown are for the progression model. The severity scores reference group is the minimal score group, therefore the figures shown are the values of each severity group compared to the minimal group.

In terms of outcomes other than recovery, e.g. reliable improvement, no change or deterioration, there was either no significance with either model type, or where there was significance it was with the stratified model.

## Discussion

In a large IAPT service collecting data on more than 16000 patients that had completed treatment according to IAPT KPI definitions, it was possible to use retrospective cohort analysis to compare outcomes in the two stepped care models due to the natural experiment opportunity of a changed service model during the period.

Proportionally the progression model had more clinician defined treatment completers, as referral rates rose, than with an earlier year using a stratified model. A confounder could be natural improvement in efficiency within the two year period, increasing turnover, volume of completed treatment, which could have had an effect on the recovery outcome increase. Whilst possible, an equally unmeasured variable that counters this possibility is that during the same period, the service experienced around 15% average annual workforce capacity reduction through maternity leave, delays in recruitment, long term sickness, and attrition. This resulted in recruiting a larger than predicted number of trainees with naturally a lower caseload capacity and potential skill. Despite this, higher recovery rates have been achieved and we have shown that the progression model is associated with higher recovery rates compared to the stratified model.

It is possible that the specific standardisation and therefore less variation of certain step 2 interventions within this service has contributed to the increase of recovery rates, thus the factor being fidelity to treatment protocol. At a higher intensity level, it is possible that model drift may be more likely to occur, with a tendency of therapists to lean more towards a transdiagnostic model with perceived complex cases. Certainly, one large IAPT study [15] states that compliance with NICE treatments is a predictor for better recovery rates. This assertion appears to be derived from the dataset including a number of IAPT services, with data outlining numbers receiving NICE compliant treatments; however protocol fidelity in each service was not specifically measured. Similarly at the time of analysis in this study, treatment protocol fidelity was not a controlled variable.

### Does complexity and severity need high intensity treatment?

The underpinning belief of a stratified model is that complex and severe cases should be treated by high intensity treatment delivered by a more qualified therapist. This belief is further perpetuated with the mixed model design of stepped care within NICE guidelines. Also in studies seeking to demonstrate the effectiveness of stepped care, using a progression model definition, however building a caveat or exception for complex cases to be treated in the first instance at a higher intensity level [19].

The growth in evidence of effectiveness of low intensity interventions, even with complexity and severity [4] raise questions regarding that notion, and indeed present a compelling and cost effective alternative for services wishing to maximise the volume of people treated in an efficient, yet clinically effective manner.

This study demonstrates that in cases with higher initial scores on PHQ9, recovery is associated with the progression model where most will receive step 2 interventions only, compared to a stratified model. This may indicate that those more severely depressed actually benefit greatly from a very simple, structured treatment. The growth in evidence regarding the effectiveness of Behaviour Activation supports this notion [20]. Whilst there was a statistical significance within the stratified model in terms of reliable improvement for patients scoring moderate to severe PHQ9, this result is likely explained by the effectiveness of treatment delivered in both cohorts. As there was no difference of patient type or presentation between the model cohorts, the statistical significance of the progression model more likely to attain recovery is a more welcome result. Essentially more people attaining reliable recovery rather than reliable improvement is the aim.

The differences in the higher initial GAD 7 scores and recovery compared to PHQ9 group were not clear enough to make conclusions. A possible confounding factor could be that this particular service was maybe more skilled at treating depression. Competencies and experience were uncontrolled variables. Type of disorder treated was not a controlled variable, and it is possible that the low intensity psycho- educative treatment was too generic and standardised to effect maximum gains for some specific anxiety presentations.

Initial scores on the psychological measures used give a self-reporting indication on frequency of symptoms and they can be a major factor in consideration of intensity of treatment. It is fair to say that self-reported scores may not necessarily correspond with other clinical information, and clinical decision making is usually based on observed presentation and assessment as well as psychological measures. This study did not account for the potential variable of presentation and scores, therefore this may be a confounding factor to the results. It may be useful for future analysis to differentiate score and presentation congruent and incongruent cases and to see whether outcomes differ.

A further possible confounding factor may be that we did not compare the clinical outcomes of those stepped up receiving treatment at step 2 then 3 to those treated at step 2 alone, as the focus was on the service delivery model, rather than the intensity or type of treatment. It is possible that certain diagnosed specific anxiety disorders (PTSD and social anxiety) would do better with a high intensity treatment in the first instance, as is currently recommended by the National IAPT manual [6]. There is currently a gap in the evidence regarding the effectiveness of specific step 2 treatments for these disorders, and also gaps in our understanding of which specific change components within therapy work for whom.

Accepting the confounding factors potential impact on the results, this study does raise the possibility that a progression model, with cases being treated at step 2 in the first instance, may achieve better recovery rates for those scoring more severely initially than those within a stratified model. This supports other studies that found no difference in the initial score severity between those treated at low or high intensity [11], or scores not to be an influencing factor on a service achieving low or high recovery rate [12]. One systematic review [10] found considerable variation of patient severity and symptom chronicity and no clear trends that related chronicity/severity to clinical outcome. One of the concerns treating patients that score highly on the measures with a low intensity intervention in the first instance could be that more patients would drop out. This study did not find this, on the contrary, dropout rate decreased in the progression model; however it is acknowledged that a possible influencing confounding factor of length of wait time prior to treatment was not controlled.

### Treatment dosage

The average dosage at each step within this study is generally consistent with the National IAPT picture [15]. However it is notable that within this study with the group of patients that are stepped up the average dosage increases by about 2 sessions. However compared to a stratified model the service achieves a bigger throughput of patients with a progression model, and the larger volume receiving step 2 only, therefore the gains are made here in terms of throughput, which in turn reduces waiting time, with less demand on the step 3 level that has the slower turnover of patient volume. Given that not enough is known regarding optimal dosage, this further supports the notion and definition of pure stepped care to commence a treatment with the least intrusive, smallest dosage first.

### Limitations

Further to the potential confounding factors acknowledged earlier, the limitations of an observational study within routine practice compared to a gold standard RCT are acknowledged. Although arguably more applicable to IAPT given the real data in a ‘real’ service, it is accepted that there are a number of uncontrolled variables that may have impacted on outcomes. There is a potential limitation to the results not controlling for effect of treatment type, however this was not the research question focus, and generally there is an acceptance of existing evidence of efficacy of treatment as recommended by NICE. Measuring the variables of disorder and treatment type however would have been useful to see if there were in fact, any difference in outcomes for PTSD and social anxiety within service model type, however as discussed previously this was not seen as viable at this point to give a meaningful result. Methodological design controlled certain variables, e.g. the cohort chosen, and given the nature of staff turnover and a proportion of continuation of trainees impacting on capacity and skill level, it is proposed that this most likely countered any effect of service improvement occurring naturally over time. Reversing the referencing groups and repeating regression tests were not undertaken, nor were more advanced regression models to test the stability of the data, and this is an accepted statistical analysis limitation.

This is an observational study using data from one service, and therefore it is possible that the results may be unique to that particular service. However the descriptive analyses show that the demographics of participants in this study are consistent with the larger national IAPT dataset [18]. Similarly results support other studies findings in terms of the efficacy of low intensity interventions even with those scoring as severe.

### Implications and challenges for practice

The study has several implications both broad and specific. Firstly, the questions raised by the difference between the stepped care model research definition and NICE guidelines, stepped care implementation within research trials and implementation in routine practice, demonstrate the need for consistency, in order to measure effectiveness of what is stated is being measured.

Second, where there is flexibility in a service delivery design, or within guidelines, it is suggested that clinicians will continue to make decisions based on the belief that complexity and severity require more intense treatment, despite a growth of evidence including this study that question the need, unless policy, guidelines and systems persuade otherwise.

Although one study shows that initial severity predicted reliable recovery, i.e. the more severe the scores the less likely to recover [15],this study shows that even those with severe scores may do better in a progression model of stepped care, where the majority of patients will receive a step 2 intervention only. Therefore whilst severity might predict recovery, it does not necessarily predict the need for high intensity treatment. The growth in the use of low intensity interventions over the past 10 years is particularly supported by the national IAPT programme. There is sound evidence of their effectiveness with depression and anxiety, and although NICE currently recommend high intensity for PTSD and social anxiety, following these guidelines with large step 3 waiting lists may leave these patients disadvantaged, and without treatment for a long period of time. Aside from perhaps the need for increased resources, it also highlights the need for improved low intensity /stabilisation treatments for these disorders that can be offered in the first instance. The rationale of the progression model is a pragmatic one; some psycho-education treatment in the first instance is better than no treatment at all. Particularly to enable services to risk manage, rather than patients sitting on waiting lists, and may promote better engagement. Although this study found a reduction in dropout rates since the progression model was introduced, we do not know enough about why patients drop out of treatment, and how specifically an increase in low intensity treatments offered in the first instance to a larger volume of patients in other services may affect dropout rates. Pragmatically, the progression model facilitates a notion that a service that can increase throughput and volume of patients treated, and thus treat more people faster, will increase engagement and completion of treatment and conversely reduce dropout.

The IAPT programme is tasked with improving access, increasing the volume of patients treated whilst delivering evidence based interventions and achieving good recovery rates. The challenge for IAPT programmes is how to achieve all three elements simultaneously.

The evidence base is changing, with the effectiveness of low intensity interventions to be demonstrable. Building on a body of evidence regarding Behavioural Activation, one study reported that BA was found to be not inferior to CBT “in terms of reduction of depression symptoms” and more cost effective [20]. Unfortunately there is little understanding in routine practice how much clinician bias and preference may influence and skew demand on levels of treatment intensity. Perhaps the biggest implication and challenge for practice, is to have confidence to use a system design to control the variable of clinician bias. A progression model of stepped care, removes this variable. In the current context of gaps in evidence regarding what specifically works for whom, a more attractive option could be where the system controls certain variables. A system which controls delivery of evidenced based low intensity interventions in the first instance, which can increase the number of patients treated, reduce costs through a larger volume being treated with a shorter number of sessions, and achieving good recovery rates may be preferable. The challenges for services are the acceptability of this system by clinicians, and the culture change process that has to occur for its success.

## Supporting information

S1 Data (XLSX)

## References

[1] Department of Health. (2008). Improving access to psychological therapies (IAPT) commissioning toolkit. Department of Health. Retrieved from http://www.iapt.nhs.uk

[2] Layard R. (2006). The depression report: A new deal for depression and anxiety disorders. Centre for Economic Performance, LSE retrieved from http://cep.lse.ac.uk/pubs/download/special/depressionreport.pdf

[3] Layard R. Clark D. Knapp M. & Mayraz G. (2007) Cost-benefit analysis of psychological therapy. National Institute Economic Review. 10 1;202(1):90–8. https://doi.org/10.1177%2F0027950107086171

[4] Bower P., Kontopantelis E., Sutton A., Kendrick T., Richards D. A., Gilbody S., & & Meyer B. (2013). Influence of initial severity of depression on effectiveness of low intensity interventions: meta-analysis of individual patient data. Bmj, 346, f540 10.1136/bmj.f540 23444423 PMC3582703

[5] National Institute for Health and Clinical Excellence (NICE) (2011)common mental health disorders identification and pathways to care retrieved from https://www.nice.org.uk/guidance/cg123 31877005

[6] National Collaborating Centre for Mental Health. (2018). The improving access to psychological therapies manual. Retrieved from https://www.england.nhs.uk/wp-content/uploads/2018/06/the-iapt-manual.pdf

[7] Bower P., & Gilbody S. (2005). Stepped care in psychological therapies: access, effectiveness and efficiency: narrative literature review. The British Journal of Psychiatry, 186(1), 11–17. 10.1192/bjp.186.1.11 15630118

[8] Richards D. A., Bower P., Pagel C., Weaver A., Utley M., Cape J., & & Owens L. (2012). Delivering stepped care: an analysis of implementation in routine practice. Implementation Science, 7(1), 3 10.1186/1748-5908-7-3 22248385 PMC3283464

[9] van Straten A., Hill J., Richards D. A., & Cuijpers P. (2015). Stepped care treatment delivery for depression: a systematic review and meta-analysis. Psychological medicine, 45(2), 231–246. 10.1017/S0033291714000701 25065653

[10] Firth N., Barkham M., & Kellett S. (2015). The clinical effectiveness of stepped care systems for depression in working age adults: a systematic review. Journal of Affective Disorders, 170, 119–130. 10.1016/j.jad.2014.08.030 25240141

[11] Chan S. W., & Adams M. (2014). Service use, drop-out rate and clinical outcomes: a comparison between high and low intensity treatments in an IAPT Service. Behavioural and cognitive psychotherapy, 42(6), 747–759. 10.1017/S1352465813000544 24382088

[12] Vaillancourt K., Manley J., & McNulty N. (2015). Why has our recovery rate dropped? An audit examining waiting times, starting scores and length of treatment in relation to recovery within an IAPT service. The Cognitive Behaviour Therapist, 8 10.1017/S1754470X15000148

[13] Barth J., Munder T., Gerger H., Nüesch E., Trelle S., Znoj H., & & Cuijpers P. (2013). Comparative efficacy of seven psychotherapeutic interventions for patients with depression: a network meta-analysis. Focus, 14(2), 229–243. 10.1371/journal.pmed.1001454 PMC6519647 31997951

[14] Department of Health. (2011). Improving access to psychological therapies (IAPT) The IAPT Data Handbook. Department of Health Retrieved from http://www.iapt.nhs.uk

[15] Gyani A., Shafran R., Layard R., & Clark D. M. (2013). Enhancing recovery rates: lessons from year one of IAPT. Behaviour Research and Therapy, 51(9), 597–606. 10.1016/j.brat.2013.06.004 23872702 PMC3776229

[16] Kroenke K., Spitzer R. L., & Williams J. B. (2001). The PHQ‐9: validity of a brief depression severity measure. Journal of general internal medicine, 16(9), 606–613. 10.1046/j.1525-1497.2001.016009606.x 11556941 PMC1495268

[17] Spitzer R. L., Kroenke K., Williams J. B., & Löwe B. (2006). A brief measure for assessing generalized anxiety disorder: the GAD-7. Archives of internal medicine, 166(10), 1092–1097. 10.1001/archinte.166.10.1092 16717171

[18] NHS Digital. (2014). Psychological Therapies, Annual Report on the use of IAPT services: England– 2013/14 Experimental Statistics. retrieved from https://digital.nhs.uk/data-and-information/publications/statistical/psychological-therapies-annual-reports-on-the-use-of-iapt-services

[19] Van Straten A., Tiemens B., Hakkaart L., Nolen W. A., & Donker M. C. H. (2006). Stepped care vs. matched care for mood and anxiety disorders: a randomized trial in routine practice. Acta Psychiatrica Scandinavica, 113(6), 468–476. 10.1111/j.1600-0447.2005.00731.x 16677223

[20] Richards D. A., Ekers D., McMillan D., Taylor R. S., Byford S., Warren F. C., & & H. (2016). Cost and Outcome of Behavioural Activation versus Cognitive Behavioural Therapy for Depression (COBRA): a randomised, controlled, non-inferiority trial. The Lancet, 388(10047), 871–880. 10.1016/S0140-6736(16)31140-0 PMC5007415 27461440

